# Clinical, psychological and brain imaging investigation of first episode psychosis patients treated at Semmelweis University, Department of Psychiatry and Psychotherapy, Budapest, Hungary

**DOI:** 10.1192/j.eurpsy.2024.612

**Published:** 2024-08-27

**Authors:** R. I. Zsigmond, L. Hermán, V. Simon, G. Csukly, E. Vass, M. Baradits, J. Réthelyi

**Affiliations:** ^1^Semmelweis University, Budapest, Hungary

## Abstract

**Introduction:**

First episode psychosis (FEP) is the first manifestation of psychotic disorders lasting at least one week, but not longer than 2 years, causing personal suffering and decreased functional outcome of patients. The early intervention in FEP is crucial. Published results on early intervention programmes indicate that during the first 5-10 years relapse prevention and functional outcomes can be improved and mental health care costs can be reduced, compared to treatment as usual.

**Objectives:**

Our objective was to examine FEP patients at the Department of Psychiatry and Psychotherapy. Our aim was to create a homogeneous sample and identify factors that can help in early differential diagnosis and therapy. Our goal was to compare the neuropsychological performance and MRI results of patients and healthy controls.

**Methods:**

Male and female inpatients hospitalized at our department due to a first psychotic episode and consenting to participate were included, since 2019 October. Cases with drug induced psychosis and organic background in the etiology of the psychotic episode were excluded. Male and female healthy controls were matched by age and education. Including healthy controls is still in progress. The duration of the project is 36 months, 24 months for recruiting patients and healthy controls, 12 month for analyzing data. The investigation includes detailed clinical, neuropsychological examination (baseline, 6th, 12th, 18th, 24th month) and MRI (baseline and in the 24th month).

**Results:**

Forty patients and sixteen healthy controls were included. 60% of the patients were rehospitalized due to relapses. Neuropsychological tests (RBANS, faux pas, Baron-Cohen eyes test) indicate cognitive dysfunction compared to healthy subjects. Using resting state fMRI second level analysis we found alterations in thalamo-cortical connectivity. We found significant differences in the connectivity of the thalamus and frontal lobe, postcentral gyrus, insula and cerebellum.

**Conclusions:**

Our FEP research, although limited by the COVID-19 pandemic, shows promising results that can help in better understanding of the underlying factors of psychotic disorders.

**Disclosure of Interest:**

None Declared

